# Does pulse pressure variation predict fluid responsiveness in critically ill patients? A systematic review and meta-analysis

**DOI:** 10.1186/s13054-014-0650-6

**Published:** 2014-11-27

**Authors:** Xiaobo Yang, Bin Du

**Affiliations:** Medical ICU, Peking Union Medical College Hospital, Peking Union Medical College and Chinese Academy of Medical Sciences, 1 Shuai Fu Yuan, Beijing, 100730 PR China

## Abstract

**Introduction:**

Fluid resuscitation is crucial in managing hemodynamically unstable patients. The last decade witnessed the use of pulse pressure variation (PPV) to predict fluid responsiveness. However, as far as we know, no systematic review and meta-analysis has been carried out to evaluate the value of PPV in predicting fluid responsiveness specifically upon patients admitted into intensive care units.

**Methods:**

We searched MEDLINE and EMBASE and included clinical trials that evaluated the association between PPV and fluid responsiveness after fluid challenge in mechanically ventilated patients in intensive care units. Data were synthesized using an exact binomial rendition of the bivariate mixed-effects regression model modified for synthesis of diagnostic test data.

**Result:**

Twenty-two studies with 807 mechanically ventilated patients with tidal volume more than 8 ml/kg and without spontaneous breathing and cardiac arrhythmia were included, and 465 were responders (58%). The pooled sensitivity was 0.88 (95% confidence interval (CI) 0.81 to 0.92) and pooled specificity was 0.89 (95% CI 0.84 to 0.92). A summary receiver operating characteristic curve yielded an area under the curve of 0.94 (95% CI 0.91 to 0.95). A significant threshold effect was identified.

**Conclusions:**

PPV predicts fluid responsiveness accurately in mechanically ventilated patients with relative large tidal volume and without spontaneous breathing and cardiac arrhythmia.

**Electronic supplementary material:**

The online version of this article (doi:10.1186/s13054-014-0650-6) contains supplementary material, which is available to authorized users.

## Introduction

Despite the fact that fluid resuscitation is considered the first line of therapy in hemodynamically unstable patients [1], a large body of evidence indicates that unnecessary positive fluid balance is associated with increased morbidity and mortality [2-4]. Moreover, conflicting results have been reported with regards to studies investigating goal-directed hemodynamic therapy in critically ill patients, such as Rivers and colleagues’ study and the OPTIMISE study [5,6]. No matter what the study result is, the first step in hemodynamic therapy in all of the above studies is always preload optimization. However, only about one-half of the critically ill patients exhibit a positive response to fluid challenge [7], and accurate prediction of fluid responsiveness remains one of the most difficult tasks at the bedside in the ICU.

Medical history, clinical manifestations (such as skin turgor, blood pressure, pulse rate, and urine output), and routine laboratory tests are important but of limited sensitivity and specificity [7,8]. Static measures, including central venous pressure, pulmonary capillary wedge pressure, right ventricular end-diastolic volume, left ventricular end-diastolic area, inferior vena-caval diameter, and global end-diastolic volume index, are of poor value in guiding fluid resuscitation [1,7,9].

Over the last decade, a number of studies have been reported, which have used heart–lung interactions during mechanical ventilation to assess fluid responsiveness. Among these functional hemodynamic parameters, pulse pressure variation (PPV) – which can easily and accurately obtained by online assessment of the arterial waveform with a standard multiparametric monitor [10] – has been shown to be highly predictive of fluid responsiveness in a systematic review, with sensitivity, specificity, and diagnostic odds ratio of 0.89, 0.88, and 59.86, respectively [11]. Nevertheless, seven out of the 29 studies included in the systematic review were performed in operating rooms rather than ICUs. In addition, more studies have been published since. The objective of this systematic review and meta-analysis was to evaluate the accuracy of PPV in predicting fluid responsiveness in ICU patients.

## Materials and methods

### Study selection and inclusion criteria

All relevant clinical trials investigating the ability of PPV to predict fluid responsiveness in adult patients in the ICU were considered for inclusion. Only studies published as full-text articles in an indexed journal were included. Reviews, case reports, and studies published in abstract form were excluded. No language restriction was applied. No ethical approval and patient consent are required.

We included those studies in which the predictive value had been assessed by calculating both sensitivity and specificity in identifying those patients who subsequently responded to fluid challenge. We excluded studies employing a ventilatory strategy that maintained spontaneous breathing or generated a tidal volume <8 ml/kg. However, those studies without clear statement concerning the presence or absence of spontaneous breathing and tidal volume were included in the final analysis.

### Search strategy and data extraction

Two authors independently performed a search in MEDLINE and EMBASE published from inception to 7 May 2014. If discrepancy existed between the two authors, it was resolved by discussion. An advanced search from the EMBASE website was used [12], with key words of pulse pressure (explode) and fluid responsiveness or fluid challenge or fluid resuscitation (explode). In addition to the electronic search, we checked out cross-references from original articles and reviews.

The two authors independently performed the initial selection by screening titles and abstracts. Citations were selected for further evaluation if the studies they referred to were studies investigating the predictive value of PPV in ICU adult patients. For detailed evaluation, we obtained peer-reviewed full texts of all possibly relevant studies. Data from each study were extracted independently using a standardized form, which included first author, year of publication, sample size, study setting, patient population, primary diagnosis, tidal volume, absence of spontaneous breathing or arrhythmia, type and amount of fluid infused, duration of fluid challenge, definition of responders, instrument(s) used for measuring index, and cardiac output. We also collected data about the method used to measure PPV as well as the threshold of PPV to achieve corresponding sensitivity and specificity. True positive, false positive, false negative, and true negative values were calculated to construct the 2 × 2 contingency table.

When two methods were compared, only the data of the reference method for measuring PPV were included. If data provided in the original study were inadequate to generate the contingency table, we sent two emails, at a 1-week interval, to the corresponding author for clarification. If the author failed to respond to our emails, we excluded the study from the final analysis.

### Quality assessment

Study quality was assessed using the Quality Assessment of Diagnostic Accuracy included in Systematic Reviews (QUADAS-2) checklist [13], an improved, redesigned tool based both on experience using the original tool [14] and new evidence about sources of bias and variation in diagnostic accuracy studies. QUADAS-2 comprised four domains: patient selection, index test, reference standard, and flow and timing. Each domain was assessed in terms of risk of bias, and the first three domains were also assessed in terms of concerns regarding applicability. Each domain was scored as ‘yes’, ‘no’, or ‘unclear’. We did not calculate summary scores because their interpretation was problematic and potentially misleading [15].

### Statistical analysis

Data were synthesized using an exact binomial rendition of the bivariate mixed-effects regression model modified for synthesis of diagnostic test data [16-18]. Theoretically, bivariate models are motivated and allow estimation of the correlation of sensitivity and specificity, because, unlike univariate analyses, bivariate models do not transform pairs of sensitivity and specificity into single indicators as diagnostic accuracy [19]. The overall pooling of sensitivity, specificity, positive likelihood ratio, negative likelihood ratio, and diagnostic odds ratio was calculated using a random-effects model. Weighted summary receiver operating characteristic (SROC) analysis was implemented. The area under the curve (AUC) with 95% confidence interval (CI) was calculated. Bayes nomogram was also constructed to show the diagnostic accuracy of PPV in prediction of fluid responsiveness.

Heterogeneity across studies was assessed using the chi-square test and the Cochran *Q* statistic was calculated. The effect of heterogeneity was quantified using inconsistency (*I*^2^), which described the percentage of total variation attributed to heterogeneity rather than chance. *I*^2^ > 50% would mean significant heterogeneity [20]. A Galbraith plot was used to identify outliers. After removal of outliers, heterogeneity test was performed as mentioned before and AUC was also recalculated.

If significant heterogeneity was recorded, the proportion probably due to the threshold effect was evaluated using the Moses–Shapiro–Littenberg method [21]. Another potential source of heterogeneity was investigated by meta-regression analysis if heterogeneity among studies could not be fully explained by the threshold effect. Publication bias was investigated using Deeks’ funnel plot asymmetry test and the *P* value for the slope coefficient was reported [22].

A subgroup analysis was carried out on different types of fluid that were used to assess fluid responsiveness. For studies reporting both the predictive value of PPV and stroke volume variation (SVV), the SROC AUC was calculated for both parameters.

Data were analyzed using STATA version 13.0 (Stata Corp LP, College Station, TX, USA) with the MIDAS module. Most data are expressed as value (95% CI). *P* <0.05 was considered statistically significant.

## Results

**Figure 1:**
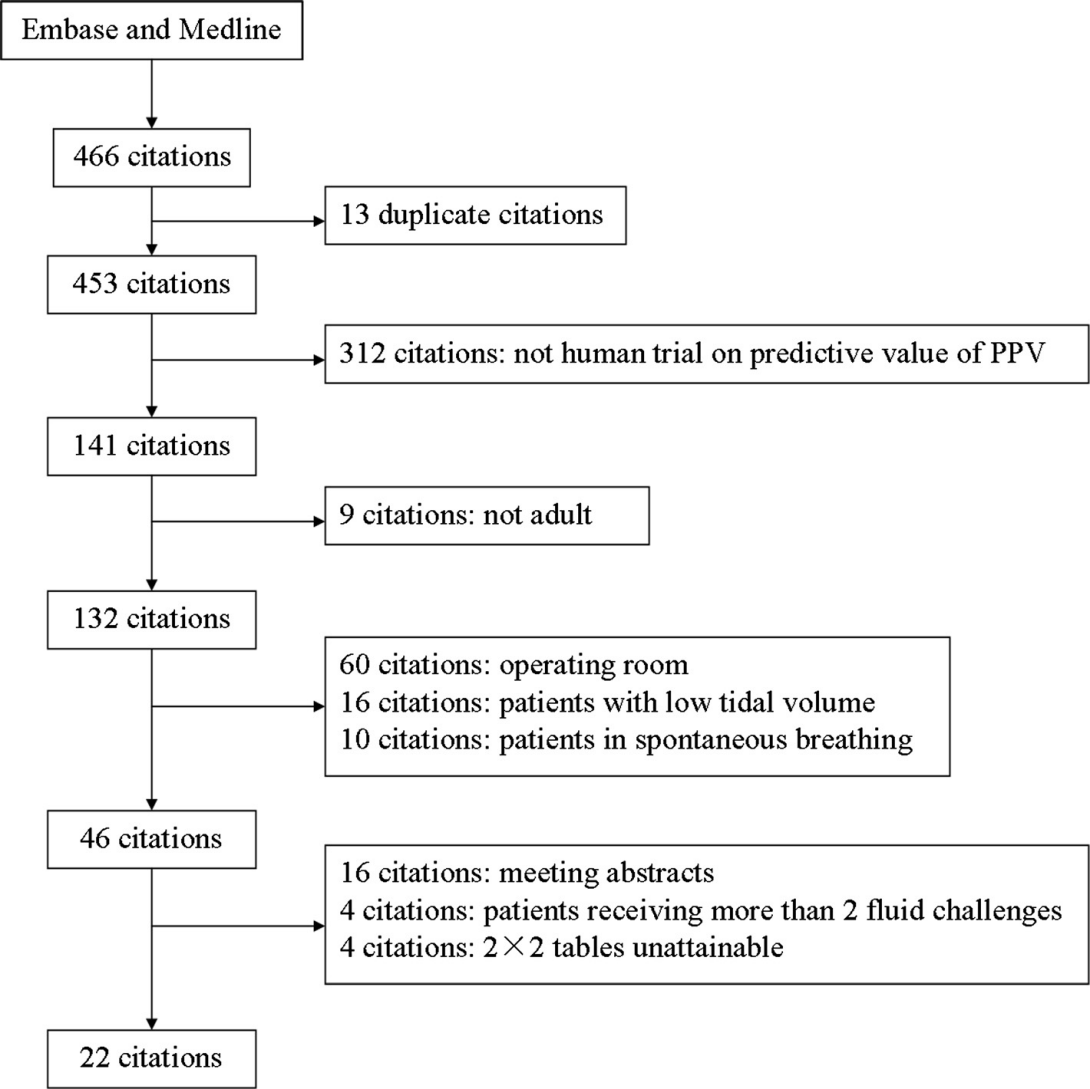
**Flowchart of study selection.** PPV, pulse pressure variation.

The process of the study search and inclusion is summarized in Figure 1. The 22 studies included in the final meta-analysis enrolled a total of 807 patients, with an average of 37 patients per study [23-44].

Characteristics of the 22 included studies are summarized in Tables 1 and 2. A total of 465 patients (58%) were responders to fluid challenge, ranging from 29 to 84% among 22 studies. Ten studies were performed in patients after cardiac surgery (*n* = 8) or other surgery (*n* = 2), while other studies were conducted in a mixed population of critically ill patients. Data on tidal volume, spontaneous breathing, and body weight were not available in some studies (Table 2). Most studies did not report the respiratory rate (Table 2). All of the included studies excluded patients with cardiac arrhythmia. With regards to fluid challenge, 6% hydroxyethyl starch (HES) was most commonly used (*n* = 13), followed by crystalloids (*n* = 6), other colloids (*n* = 2), and blood (*n* = 1). A volume ranging from 100 ml to 20 ml/kg body weight was infused within 1 minute up to 90 minutes. Cardiac output was monitored by means of PiCCO technology (*n* = 7), pulmonary artery catheter (*n* = 6), transesophageal/transthoracic echocardiography or Doppler measurements (*n* = 5), and other methods (*n* = 4). All studies except for two [24,26] defined responders as patients whose stroke volume or cardiac output was increased by ≥15%.

**Table 1:** **Selected spectrum characteristics of included studies**

**Order**	**Authors**	**Year**	**Sample size**	**Setting**	**Admission**	**Diagnosis**
1	Michard and colleagues [23]	2000	40	ICU	Medical	Sepsis/septic shock
2	Kramer and colleagues [24]	2004	21	SICU	Surgical	Cardiac surgery
3	Feissel and colleagues [25]	2005	20^a^	ICU	Medical	Sepsis/septic shock
4	Charron and colleagues [26]	2006	21	ICU	NA	NA
5	Monnet and colleagues [27]	2006	30	MICU	Medical	Shock
6	Feissel and colleagues [28]	2007	23^b^	MICU	Medical	Sepsis/septic shock
7	Wyffels and colleagues [29]	2007	32	ICU	Surgical	Cardiac surgery
8	Auler and colleagues [30]	2008	59	SICU	Surgical	Cardiac surgery
9	Monge Garcia and colleagues [31]	2009	38	ICU	NA	Shock
10	Vistisen and colleagues [32]	2009	23	SICU	Surgical	Cardiac surgery
11	Loupec and colleagues [33]	2011	40	SICU	Surgical and medical	Surgery and septic shock
12	Biais and colleagues [34]	2012	35	SICU	Surgical and medical	Vascular surgery, trauma, septic shock
13	Cecconi and colleagues [35]	2012	31	ICU	Surgical	High-risk surgery
14	Fellahi and colleagues [36]	2012	25	SICU	Surgical	Cardiac surgery
15	Khwannimit and colleagues [37]	2012	42	MICU	Medical	Sepsis/septic shock
16	Monnet and colleagues [38]	2012	26	MICU	Medical	Shock
17	Monnet and colleagues [39]	2012	39	MICU	Medical	Shock
18	Yazigi and colleagues [40]	2012	60	SICU	Surgical	Cardiac surgery
19	Fischer and colleagues [41]	2013	37	SICU	Surgical	Cardiac surgery
20	Fischer and colleagues [42]	2013	80	SICU	Surgical	Cardiac surgery
21	Ishihara and colleagues [43]	2013	43	ICU	Surgical	Noncardiac surgery
22	Monnet and colleagues [44]	2013	35	MICU	Medical	Shock

MICU, medical intensive care unit; NA, not available; SICU, surgical intensive care unit. ^a^Twenty-two fluid challenges included. ^b^Twenty-eight fluid challenges included.

**Table 2:** **Selected methodological characteristics of included studies**

**Order**	**Authors**	**Year**	**Vt (ml/kg)**	**Body weight**	**Spontaneous breathing**	**Respiratory rate (times/minute)**	**Cardiac arrhythmia**	**Infusion fluid**	**Infusion volume**	**Infusion time (minutes)**	**Indices**	**Method for indices**	**Cutoff value (%)**
1	Michard and colleagues [23]	2000	8 to 12	NA	No	NA	No	6% HES	500 ml	30	CI	PAC	15
2	Kramer and colleagues [24]	2004	8 to 10	NA	No	NA	No	Blood	500 ml	10 to 15	CO	PAC	12
3	Feissel and colleagues [25]	2005	8 to 10	NA	No	NA	No	6% HES	7 ml/kg	30	CI	Echo	15
4	Charron and colleagues [26]	2006	6 to 10	NA	No	14 to 20	No	6% HES	100 ml	1	SV	Echo	15
5	Monnet and colleagues [27]	2006	NA	NA	No	23 ± 5	No	Saline	500 ml	10	Aortic blood flow	Esophageal Doppler	15
6	Feissel and colleagues [28]	2007	8 to 10	NA	No	NA	No	6% HES	8 ml/kg	30	CI	Echo	15
7	Wyffels and colleagues [29]	2007	8 to 10	NA	No	NA	No	6% HES	500 ml	20	CO	PAC	15
8	Auler and colleagues [30]	2008	8	NA	No	NA	No	LR	20 ml/kg	20	CI	PAC	15
9	Monge Garcia and colleagues [31]	2009	9	Ideal	No	18 to 20	No	6% HES	500 ml	30	SV	FloTrac	15
10	Vistisen and colleagues [32]	2009	8.1^a^	Predicted	NA	14	No	6% HES	500 ml	45	CI	PAC	15
11	Loupec and colleagues [33]	2011	8 to 10	Predicted	No	NA	No	6% HES^b^	500 ml	10	CO	Echo	15
12	Biais and colleagues [34]	2012	8 to 10	Predicted	No	16 ± 3^c^	No	Saline	500 ml	15	SV	Echo	15
13	Cecconi and colleagues [35]	2012	8	Ideal	No	14	No	Colloid	250 ml	5	CO	LiDCO plus	15
14	Fellahi and colleagues [36]	2012	NA	NA	NA	12 ± 2	No	6% HES	500 ml	15	CI	PiCCO2	15
15	Khwannimit and colleagues [37]	2012	≥ 8	NA	No	NA	No	6% HES	500 ml	30	SV	FloTrac	15
16	Monnet and colleagues [38]	2012	8.8^d^	Predicted	No	NA	No	Saline	500 ml	20	CI	PiCCO2	15
17	Monnet and colleagues [39]	2012	8.5^e^	Predicted	No	NA	No	Saline	500 ml	30	CI	PiCCO2	15
18	Yazigi and colleagues [40]	2012	8	NA	No	12	No	6% HES	7 ml/kg	20	SV	PAC	15
19	Fischer and colleagues [41]	2013	8.6	NA	NA	NA^f^	No	6% HES	500 ml	15	CI	PiCCO2	15
20	Fischer and colleagues [42]	2013	8.2	NA	No	NA^c^	No	6% HES	500 ml	15	CI	PiCCO2	15
21	Ishihara and colleagues [43]	2013	≥ 8	Ideal	No	12 to 15	No	10% dextran	250 ml	20	CI	PiCCO	15
22	Monnet and colleagues [44]	2013	9	Predicted	No	NA	No	Saline	500 ml	30	CI	PiCCO2	15

CI, cardiac index; CO, cardiac output; Echo, echocardiography; HES, hydroxyethyl starch; LR, Ringer’s lactate; NA, not available; PAC, pulmonary artery catheter; SV, stroke volume; Vt, tidal volume. ^a^Mean Vt of 6.9 ml/kg actual body weight. ^b^Some patients evaluated with passive leg raising test. ^c^Heart rate/respiratory rate >3.6. ^d^Only mean Vt >8 ml/kg included. ^e^Mean Vt of 7.4 ml/kg for nonresponder. ^f^Heart rate/respiratory rate = 5.7.

Overall, the methodological quality was moderate (Additional file 1). Specifically, five out of 22 included studies reported enrollment of consecutive patients, and four studies reported no test review bias; that is, interpretation of the reference standard results without knowledge of the result of the index test, and *vice versa*.

The results for the diagnostic performance of the included studies and the method used to measure PPV are presented in Table 3. The median threshold of PPV in predicting fluid responsiveness was 12% (interquartile range 10 to 13%).

**Table 3:** **Diagnostic performance of pulse pressure variation from included studies**

**Order**	**Authors**	**Year**	**Threshold** ^**a**^ **(%)**	**tp**	**fp**	**fn**	**tn**	**Sens. (%)**	**Spec. (%)**	**Method used to measure PPV**
1	Michard and colleagues [23]	2000	13	15	1	1	23	94	96	Waveform analysis with computer software
2	Kramer and colleagues [24]	2004	11	6	1	0	14	100	93	Waveform analysis with computer software
3	Feissel and colleagues [25]	2005	17	11	0	2	9	85	100	Waveform analysis with computer software
4	Charron and colleagues [26]	2006	10	8	2	1	10	89	83	Waveform analysis with computer software
5	Monnet and colleagues [27]	2006	12	14	1	2	13	88	93	Waveform analysis with computer software
6	Feissel and colleagues [28]	2007	12	18	1	0	9	100	94	Waveform analysis with computer software
7	Wyffels and colleagues [29]	2007	11.3	19	1	1	11	95	92	Analysis of printout curves
8	Auler and colleagues [30]	2008	12	38	1	1	19	97	95	Waveform analysis with computer software
9	Monge Garcia and colleagues [31]	2009	10	18	1	1	18	95	95	Waveform analysis with computer software
10	Vistisen and colleagues [32]	2009	6.5	16	1	1	5	94	83	Waveform analysis with computer software
11	Loupec and colleagues [33]	2011	13	19	2	2	17	90	89	Waveform analysis with computer software
12	Biais and colleagues [34]	2012	10	17	2	2	14	89	88	Waveform analysis with computer software
13	Cecconi and colleagues [35]	2012	13	10	5	2	14	83	74	Waveform analysis with LiDCO
14	Fellahi and colleagues [36]	2012	10	17	1	4	3	81	75	Waveform analysis with PiCCO
15	Khwannimit and colleagues [37]	2012	12	20	3	4	15	83	83	Direct analysis of monitored arterial tracing
16	Monnet and colleagues [38]	2012	12	13	0	2	11	85	100	Waveform analysis with PiCCO
17	Monnet and colleagues [39]	2012	10	15	2	2	20	88	91	Waveform analysis with PiCCO
18	Yazigi and colleagues [40]	2012	11.5	33	5	8	14	80	74	Direct analysis of monitored arterial tracing
19	Fischer and colleagues [41]	2013	16	12	0	15	10	44	100	Waveform analysis with PiCCO
20	Fischer and colleagues [42]	2013	14	36	5	21	18	64	78	Waveform analysis with PiCCO
21	Ishihara and colleagues [43]	2013	8.5	11	6	12	14	50	71	Waveform analysis with PiCCO
22	Monnet and colleagues [44]	2013	15	14	1	1	19	93	95	Waveform analysis with PiCCO

fn, false negative; fp, false positive; PPV, pulse pressure variation; sens., sensitivity; spec., specificity; tn, true negative; tp, true positive. ^a^Threshold used in studies to achieve corresponding sensitivity and specificity.

**Figure 2:**
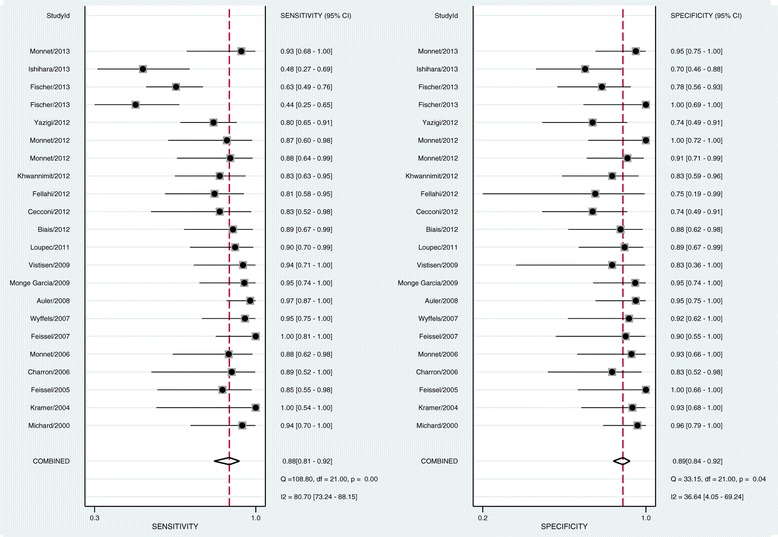
**Sensitivity and specificity of pulse pressure variation for prediction of fluid responsiveness.** CI, confidence interval.

**Figure 3:**
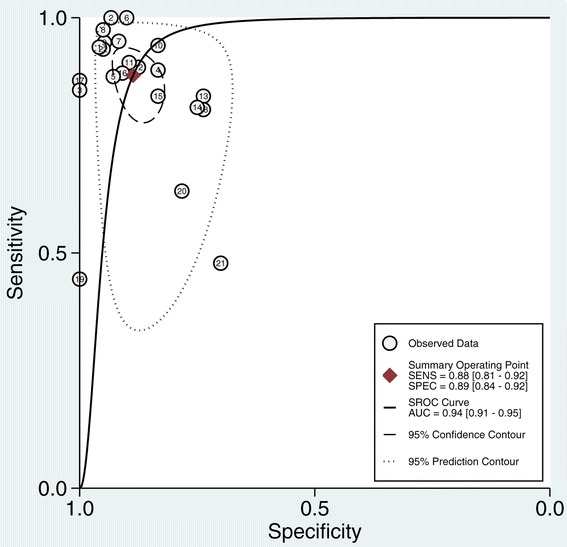
**Summary receiver operating characteristic curve.** AUC, area under the curve; SENS, sensitivity; SPEC, specificity; SROC, summary receiver operating characteristic.

Pooled sensitivity was 0.88 (95% CI = 0.81 to 0.92) and pooled specificity was 0.89 (95% CI = 0.84 to 0.92) (Figure 2). PPV had an overall positive likelihood ratio of 7.7 (95% CI = 5.3 to 11.3), a negative likelihood ratio of 0.14 (95% CI = 0.08 to 0.23), and a diagnostic odds ratio of 56 (95% CI = 26 to 122). The SROC AUC was 0.94 (95% CI = 0.91 to 0.95) (Figure 3).

**Figure 4:**
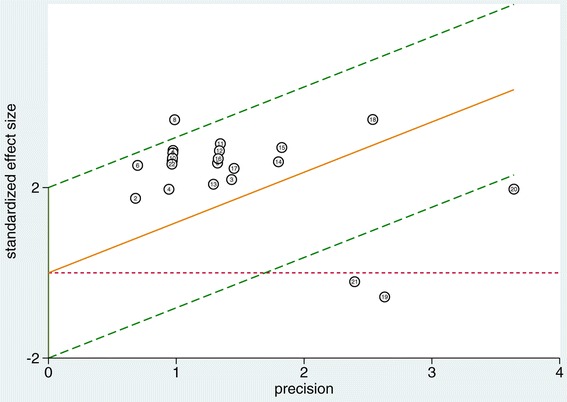
**Outliers identified using a Galbraith plot.**

Moderate heterogeneity existed among the studies, with Cochran *Q* statistic of 4.681 (*P* = 0.042) and overall *I*^2^ for bivariate model of 60% (95% CI = 9 to 100). All of the heterogeneity (100%) was caused by the threshold effect. As a result, meta-regression analysis with the objective to explore the effects of potential covariates on the diagnostic performance of PPV was not performed. Four outliers were identified by means of a Galbraith plot (Figure 4) [30,41-43]. Heterogeneity became nonsignificant (Cochrane *Q* statistic = 0.146, *P* = 0.465) after removal of these four outliers, while the SROC AUC slightly improved (0.95, 95% CI = 0.93 to 0.97).

**Figure 5:**
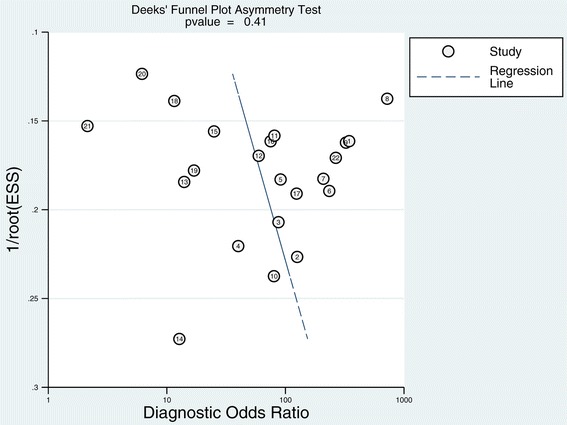
**Deeks’ funnel plot with superimposed regression line.** *P *value for slope coefficient is 0.41, which is greater than 0.05, suggesting the symmetry of the studies and the low likelihood of publication bias.

**Figure 6:**
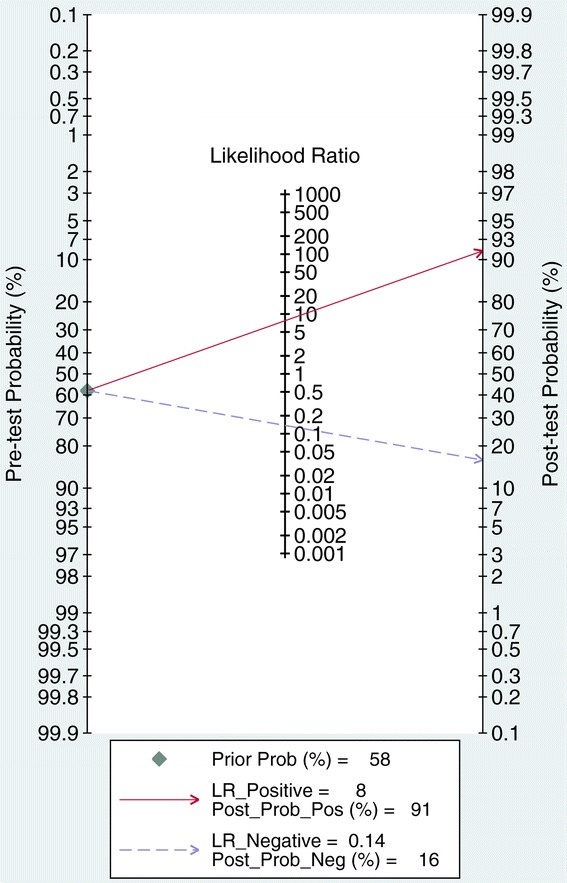
**Bayes nomogram of pulse pressure variation for prediction of fluid responsiveness.**

We did not identify publication bias by Deeks’ regression test of asymmetry; a statistically nonsignificant *P* value of 0.41 for the slope coefficient suggested symmetry in the data and a low likelihood of publication bias (Figure 5). Bayes nomogram showed moderate fine likelihood ratios and post-test probabilities (Figure 6).

For studies using HES or saline to assess fluid responsiveness, the SROC AUC of PPV was 0.92 (95% CI = 0.89 to 0.94) and 0.96 (95% CI = 0.94 to 0.98), respectively. Nine studies reported the predictive value of both PPV and SVV, and both SROC AUCs were 0.90 (95% CI = 0.87 to 0.93) (Additional file 2).

## Discussion

The major finding of this systematic review and meta-analysis is that PPV during controlled mechanical ventilation with tidal volume >8 ml/kg is an accurate predictor of fluid responsiveness in critically ill patients, with sensitivity of 0.88 (95% CI = 0.81 to 0.92), specificity of 0.89 (95% CI = 0.84 to 0.92), and SROC AUC of 0.94 (95% CI = 0.91 to 0.95).

In a meta-analysis of the literature to determine the ability of PPV, systolic pressure variation, and SVV to predict fluid responsiveness, Marik and colleagues included 29 studies enrolling a total of 685 patients and reported the pooled ROC AUC for PPV as 0.94 (95% CI = 0.93 to 0.95) [11], which is in accordance with our findings. Very recently, Hong and colleagues published another meta-analysis involving 865 mechanically ventilated patients from 19 studies, to evaluate the value of both SVV and PPV in predicting fluid responsiveness [45]. These authors reported a significantly lower pooled ROC AUC for PPV (0.88, 95% CI = 0.84 to 0.92). However, there are several major differences between these two meta-analyses and our study. First, most of the included studies in the above two meta-analyses (12 out of 18 studies [11] and 12 out of 19 studies [45], respectively) were conducted in perioperative settings (that is, before or after induction of anesthesia) or during surgery. In contrast, we limited our meta-analysis to those studies in critically ill patients in the ICU. Perioperative patients differ from critically ill patients in the ICU with regards to age, comorbidities, severity of illness, complications, and clinical outcome. Moreover, the predictive ability of PPV might be compromised in open chest conditions, due to inaccurate reflection of phasic changes in preload (and hence stroke volume), increased aortic impedance (and subsequent changes in the relationship between stroke volume and pulse pressure), as well as less pronounced cyclic changes in intrathoracic pressure [41,46]. Second, we adopted very strict inclusion and exclusion criteria in our meta-analysis. We excluded studies with a tidal volume <8 ml/kg. But one study [47] from Marik and colleagues’ review and one study [48] from Hong and colleagues’ review included tidal volume <7 ml/kg. We excluded two studies [49,50] included by Marik and colleagues because they reported PPV in fewer than 10 patients so that the 2 × 2 contingency table could not be generated. In addition, two studies with contradicting information without clarification from the authors were also excluded [51,52]. Third, Marik and colleagues did not find any heterogeneity using the Cochran *Q* statistic. Despite the fact that two parameters were meta-analyzed with only one Cochran’s *Q* statistic reported, Hong and colleagues considered between-study heterogeneity low enough to consider the study population as statistically homogeneous for applying appropriate AUC statistics. We reported borderline heterogeneity in our studies. Possible reasons might include the different studies involved in the meta-analysis (as mentioned above) as well as different statistical models used. We found that the heterogeneity observed in our meta-analysis could be fully explained by the threshold effect, which had not been investigated in the previous meta-analyses. The threshold effect is one of main causes of heterogeneity in test accuracy studies. It arises owing to different thresholds or cutoff values used in different studies to determine a positive (or negative) test result. The median threshold level for PPV to predict fluid responsiveness in our meta-analysis was 12% (interquartile range 10 to 13%) with a sensitivity of 0.88 and a specificity of 0.89. The bottom line is that we believe it would be reasonable to postulate that PPV >13% implied fluid responsiveness, while PPV <10% indicated fluid unresponsiveness.

To explore other potential sources of heterogeneity, we focused on the studies with the lowest sensitivity value and Youden index (sensitivity + specificity −1) (Figure 3). These were also three of the four studies identified using a Galbraith plot (Figure 4). Two of the studies were carried out similarly by the same first author [41,42]. In the study with larger sample size, Fischer and colleagues found that one-third of the patients had right ventricular dysfunction and 34% of ICU patients with PPV >12% were fluid unresponsive [42]. Wyler von Ballmoos and colleagues supported that right ventricular dysfunction led to poor response to fluids in patients with increased pulmonary artery pressure [53]. Similar to Fischer and colleagues’ study, Biais and colleagues evaluated right ventricular function using echocardiography and excluded patients with right ventricular dysfunction from their study [34]. Ishihara and colleagues examined the predictive value of PPV in patients after open-chest abdominothoracic esophagectomy with extensive resection of lymph nodes [43]. They postulated that extensive alteration of thoracic structure might change the cyclic variation of intrathoracic pressure, leading to a modified heart–lung interaction. Postoperative left pleural effusion was common in their patients. Right ventricular dysfunction and extensive alteration of thoracic structure will limit the use of PPV to predict fluid responsiveness.

In the OPTIMISE study, patients in the cardiac output-guided hemodynamic therapy algorithm group were given fluid challenges to reach maximal stroke volume, but no difference of a composite outcome of complications or 30-day mortality was found between the trial group and the usual care group in patients undergoing major gastrointestinal surgery [6]. The POEMAS Study found similar results [54]. However, fluid responsiveness does not necessarily mean that the patients require fluid resuscitation, as long as there are no signs of tissue hypoperfusion [55]. The cardiac functions of critically ill patients and their disease status are dynamic, while the adequacy of tissue perfusion should be the focus [56].

Our study is subject to some limitations. First, sample sizes of the included studies were small. However, even under this condition a meta-analysis still provides valuable information on the diagnostic accuracy until proven otherwise by larger or better-conducted studies [57]. Second, different instruments (pulmonary artery catheter, echocardiography, PiCCO, and so forth) and indices (cardiac output/index, stroke volume, and so forth) were used to evaluate fluid responsiveness, and different methods were used to measure PPV. Nonetheless, this reflects the real situation in a contemporary ICU. It is also convenient to evaluate PPV by directly analyzing monitored arterial tracing or printout curves [29,37,40], which means PPV can be evaluated repeatedly and regularly, and with computer software and waveform analysis can be monitored continuously. This is attractive to intensivists who have to titrate fluid resuscitation in mechanically ventilated patients almost every day. Nevertheless, it is important that elimination or minimization of fluid responsiveness, including PPV, should never be the only goal of fluid therapy. Third, we excluded studies in which patients were ventilated using low tidal volumes, which is the commonly accepted ventilation strategy in patients with acute respiratory distress syndrome. Because most studies showed that although better than conventional static parameters, PPV had limited value in predicting fluid responsiveness in these patients [52,58-64]. This was probably because, with low tidal volume ventilation, the cyclic changes in intrathoracic pressures were not significant enough to induce preload variations [58]. Huang and colleagues and Freitas and colleagues, however, found that PPV accurately predicted fluid responsiveness in patients ventilated with a tidal volume of 6 ml/kg [65,66]. They attributed the discrepancy to higher positive end-expiratory pressure levels, which enhanced the cyclic changes in pleural pressures. More trials concerning the effect of positive end-expiratory pressure on PPV are needed in the future. Before more data are presented, a temporary change of ventilator parameters maybe used to evaluate PPV at a larger tidal volume, as demonstrated by Freitas and colleagues [66]. Fourth, more than one-half of the included studies used HES. Nowadays, however, fewer patients receive HES with consideration of its detrimental effect on the kidney, as in the OPTIMISE study [6]. However, we performed a subgroup analysis and found that the SROC AUC of PPV was 0.92 (95% CI = 0.89 to 0.94) for studies using HES and 0.96 (95% CI = 0.94 to 0.98) for studies using saline, suggesting that the choice of fluid does not influence the predictive value of PPV. Fifth, ICU patients are often with conditions precluding the use of PPV. In their 1-day prospective observational study, Mahjoub and colleagues found that only 15 out of 79 (19%) patients used PPV to measure fluid responsiveness [67]. However, as advocated by Teboul and Monnet, other fluid responsiveness tests, such as passive leg raising test or end-expiratory occlusion test, could be used when PPV was not available [68]. Measurement of SVV is also influenced, as that of PPV, by the presence of cardiac arrhythmia or spontaneous breathing, as well as low tidal volume ventilation, while it requires much more complicated devices than PPV. Two meta-analyses suggested that both SVV and PPV are accurate predictors of fluid responsiveness in hemodynamically unstable patients under controlled mechanical ventilation [11,45]. With studies reporting both PPV and SVV included in our meta-analysis, we found similar results. Sixth, only Biais and colleagues and Fischer and colleagues evaluated right ventricular function using echocardiography, and only Biais and colleagues excluded patients with right ventricular dysfunction from their study [34,42].

## Conclusions

PPV is an accurate predictor of fluid responsiveness in critically ill patients passively ventilated with tidal volume >8 ml/kg and without cardiac arrhythmia.

## Key messages

- A significant threshold effect existed while using PPV to determine a responder (or nonresponder) to volume expansion.
- PPV is an accurate predictor of fluid responsiveness in critically ill patients ventilated with relative large tidal volume and without spontaneous breathing and cardiac arrhythmia.

## Additional files

Additional file 1: Table S1. Presenting a summary of QUADAS-2 quality assessment. We tailored QUADAS-2 to our review by omitting the second signalling question in domain 2′: If a threshold was used, was it pre-specified?’. Reasons for classifying high risk or unclear risk of bias are provided in footnotes for each study. Additional file 2: Table S2. Presenting the diagnostic performance of PPV and SVV from included studies reporting both parameters.

## Abbreviations

AUC: area under the curve
CI: confidence interval
HES: hydroxyethyl starch
PPV: pulse pressure variation
QUADAS: Quality Assessment of Diagnostic Accuracy included in Systematic Reviews
SROC: summary receiver operating characteristic
SVV: stroke volume variation
