# Supplementary material for: Does pulse pressure variation predict fluid responsiveness in critically ill patients? A systematic review and meta-analysis
Source: Crit Care. 2014 Nov 27;18(6):650. doi: 10.1186/s13054-014-0650-6 (PMC4258282; doi:10.1186/s13054-014-0650-6)
Supplement: Additional file 1: Table S1. — Presenting a summary of QUADAS-2 quality assessment. We tailored QUADAS-2 to our review by omitting the second signalling question in domain 2′: If a threshold was used, was it pre-specified?’. Reasons for classifying high risk or unclear risk of bias are provided in footnotes for each study. [file 13054_2014_650_MOESM1_ESM.doc]

Additional file 1: Table S1. Summary of QUADAS-2 quality assessment (reasons for classifying high risk or unclear risk of bias are provided in footnotes for each study)

| **Study** | **RISK OF BIAS** | | | | **APPLICABILITY CONCERNS** | | |
| --- | --- | --- | --- | --- | --- | --- | --- |
|  | **PATIENT SELECTION** | **INDEX TEST** | **REFERENCE STANDARD** | **FLOW AND TIMING** | **PATIENT SELECTION** | **INDEX TEST** | **REFERENCE STANDARD** |
| Michard [20] | ?^1^ | ?^2^ | ?^3^ | ☺ | ☺ | ☺ | ☺ |
| Kramer [21] | ? ^1^ | ? ^2^ | ? ^3^ | ☺ | ☺ | ☺ | ☺ |
| Feissel [22] | ? ^1^ | ? ^2^ | ? ^3^ | ☺ | ☺ | ☺ | ☺ |
| Charron [23] | ? ^1^ | ? ^2^ | ? ^3^ | ☺ | ☺ | ☺ | ☺ |
| Monnet [24] | ? ^1^ | ? ^2^ | ☹^3,4^ | ☺ | ☺ | ☺ | ? ^4^ |
| Feissel [25] | ? ^1^ | ? ^2^ | ? ^3^ | ☺ | ☺ | ☺ | ☺ |
| Wyffels [26] | ☺ | ? ^2^ | ? ^3^ | ☺ | ☺ | ☺ | ☺ |
| Auler [27] | ? ^1^ | ? ^2^ | ? ^3^ | ☺ | ☺ | ☺ | ☺ |
| Monge Garcia [28] | ? ^1^ | ? ^2^ | ? ^3^ | ☺ | ☺ | ☺ | ☺ |
| Vistisen [29] | ? ^1^ | ? ^2^ | ? ^3^ | ☺ | ☺ | ☺ | ☺ |
| Loupec [30] | ? ^1^ | ☺ | ☺ | ☺ | ☺ | ☺ | ☺ |
| Biais [31] | ? ^1^ | ☺ | ☺ | ☺ | ☺ | ☺ | ☺ |
| Cecconi [32] | ? ^1^ | ? ^2^ | ? ^3^ | ☺ | ☺ | ☺ | ☺ |
| Fellahi [33] | ? ^1^ | ? ^2^ | ? ^3^ | ☺ | ☺ | ☺ | ☺ |
| Khwannimit [34] | ? ^1^ | ? ^2^ | ? ^3^ | ☺ | ☺ | ☺ | ☺ |
| Monnet [35] | ? ^1^ | ? ^2^ | ? ^3^ | ☺ | ☺ | ☺ | ☺ |
| Monnet [36] | ? ^1^ | ☺ | ☺ | ☺ | ☺ | ☺ | ☺ |
| Yazigi [37] | ☺ | ☺ | ☺ | ☺ | ☺ | ☺ | ☺ |
| Fischer [38] | ☺ | ? ^2^ | ? ^3^ | ☺ | ☺ | ☺ | ☺ |
| Fischer [39] | ☺ | ? ^2^ | ? ^3^ | ☺ | ☺ | ☺ | ☺ |
| Ishihara [40] | ☺ | ? ^2^ | ? ^3^ | ☺ | ☺ | ☺ | ☺ |
| Monnet [41] | ? ^1^ | ? ^2^ | ? ^3^ | ☺ | ☺ | ☺ | ☺ |

☺Low Risk ☹High Risk ? Unclear Risk

We tailored QUADAS-2 to our review by omitting the second signalling question in domain 2 ’ If a threshold was used, was it pre-specified?’.

1 unclear whether a consecutive or random sample of patients enrolled; 2 unclear the index test results interpreted without knowledge of the results of the reference standard; 3 unclear whether the reference standard results interpreted without knowledge of the results of the index test; 4 aortic blood flow used as reference index.
