# Supplementary material for: Does pulse pressure variation predict fluid responsiveness in critically ill patients? A systematic review and meta-analysis
Source: Crit Care. 2014 Nov 27;18(6):650. doi: 10.1186/s13054-014-0650-6 (PMC4258282; doi:10.1186/s13054-014-0650-6)
Supplement: Additional file 2: Table S2. — Presenting the diagnostic performance of PPV and SVV from included studies reporting both parameters. [file 13054_2014_650_MOESM2_ESM.doc]

Additional file 2: Table S2. Diagnostic performance of pulse pressure variation and stroke volume variation from included studies reporting both parameters

| Order | Author | Year | Pulse Pressure Variation | | | | | | |  | Stroke Volume Variation | | | | | | |
| --- | --- | --- | --- | --- | --- | --- | --- | --- | --- | --- | --- | --- | --- | --- | --- | --- | --- |
|  |  |  | Threshold | tp | fp | fn | tn | Sen. | Spe. |  | Threshold | tp | fp | fn | tn | Sen. | Spe. |
| 1 | Monge Garcia [30] | 2009 | 10% | 18 | 1 | 1 | 18 | 95% | 95% |  | 11% | 15 | 2 | 4 | 17 | 79% | 89% |
| 2 | Biais [33] | 2012 | 10% | 17 | 2 | 2 | 14 | 89% | 88% |  | 12.6% | 12 | 5 | 7 | 11 | 63% | 69% |
| 3 | Cecconi [34] | 2012 | 13% | 10 | 5 | 2 | 14 | 83% | 74% |  | 12.5% | 9 | 3 | 3 | 16 | 75% | 83% |
| 4 | Fellahi [35] | 2012 | 10% | 17 | 1 | 4 | 3 | 81% | 75% |  | 11% | 17 | 1 | 4 | 3 | 81% | 75% |
| 5 | Khwannimit [36] | 2012 | 12% | 20 | 3 | 4 | 15 | 83% | 83% |  | 10% | 22 | 3 | 2 | 15 | 91.7% | 83.3% |
| 6 | Monnet [37] | 2012 | 12% | 13 | 0 | 2 | 11 | 85% | 100% |  | 14% | 13 | 4 | 4 | 18 | 76% | 82% |
| 7 | Fischer [40] | 2013 | 16% | 12 | 0 | 15 | 10 | 44% | 100% |  | 16% | 8 | 1 | 19 | 9 | 30% | 90% |
| 8 | Ishihara [42] | 2013 | 8.5% | 11 | 6 | 12 | 14 | 50% | 71% |  | 10.5% | 10 | 4 | 13 | 16 | 45% | 81% |
| 9 | Monnet [43] | 2013 | 15% | 14 | 1 | 1 | 19 | 93% | 95% |  | 10% | 14 | 2 | 1 | 18 | 93% | 90% |

fn: false negative; fp: false positive; sen, sensitivity; spe, specificity; tn: true negative; tp: true positive

Threshold: threshold used in studies to achieve corresponding sensitivity and specificity
